# Towards efficient AED deployment: a multi-source data-driven geographic information approach

**DOI:** 10.3389/fpubh.2026.1836037

**Published:** 2026-05-29

**Authors:** Yeling Wu, Yiji Li, Chaowei Wu

**Affiliations:** 1 Department of Orthopedics, The First Affiliated Hospital, Fujian Medical University, Fuzhou, China; 2 Department of Orthopedics, National Regional Medical Center, Binhai Campus of The First Affiliated Hospital, Fujian Medical University, Fuzhou, China; 3 Ningbo Emergency Medical Center, Ningbo, China; 4 School of Public Health, Fudan University, Shanghai, China

**Keywords:** accessibility, automated external defibrillators, geographic information model, out-of-hospital cardiac arrest, spatial deployment strategy

## Abstract

Out-of-hospital cardiac arrest (OHCA) poses a severe public health challenge, and improving the accessibility of automated external defibrillators (AEDs) is crucial for increasing survival rates. This study introduces a comprehensive spatial deployment strategy for AEDs in metropolitan areas, integrating current status assessment, multi-factor modeling, and phased planning, validated in Shanghai’s urban region. Based on Geographic Information System (GIS) software, the current status of AED accessibility was evaluated using two indicators: geographic coverage rate and population coverage rate. Subsequently, a model was constructed by integrating elements of population distribution, medical services, and the urban built environment to determine priority zones for device deployment. 92.022% of the AEDs installed before 2021 were located within the model’s recommended installation zones. Based on the model results, a three-phase device deployment plan was proposed. The completion of the first phase of the deployment plan achieved comparable service coverage to the real-world plan with a reduced number of equipment installations and about 10% more population coverage than the real-world plan. The first phase of the deployment plan expanded geographic coverage by 12.135% and increased the population served by 22.267% compared to the pre-2021 scenario. After completion of all three phases of the deployment plan, it will cover 83.653% of the geographic area and 96.486% of the population in the study area. Multi-source data-driven geographic information strategy for AED deployment strategy showed greater efficiency to better support decision makers in improving accessibility of AEDs in metropolitan areas.

## 1 Introduction

According to data from the Global Resuscitation Alliance, improving the survival rate of out-of-hospital cardiac arrest (OHCA) is an important public health goal. OHCA is an emergency medical situation that is critical and time-sensitive (1, 2), influenced by various factors such as the ability to receive timely emergency medical support (3), the patient’s physical characteristics (4), which leads to the loss of millions of lives worldwide each year (5–7).

The success of resuscitation efforts is heavily contingent upon the rapid response of bystanders and the availability of life-saving equipment (2, 8, 9), such as AEDs. Evidence from both developed and developing countries has consistently demonstrated that AEDs significantly increase survival rates from OHCA by facilitating early defibrillation (3, 10, 11). Nevertheless, the optimal deployment of AEDs is a crucial determinant of their efficacy (12, 13). It has been demonstrated that the rational deployment of AEDs can result in a significant reduction in mortality from OHCA by enhancing device accessibility (11, 14). The issue of improving access to AEDs has been identified as a significant public health concern that requires prompt attention and a strategic approach to intervention.

Given that OHCA patients require treatment within the shortest possible time frame, bystanders or rescuers typically do not need to consider restrictions imposed by administrative boundaries or complex road networks when accessing devices. In such contexts, the straight-line distance (Euclidean distance) emerges as a critical factor limiting device accessibility. From a geospatial perspective, the accessibility of AED devices is defined as the degree of difficulty in acquiring an AED, measured by distance or time. Specifically, when an OHCA event occurs at a specific location, the greater the distance between the AED device and the patient, the lower the accessibility of the AED device. This manifests as increased difficulty for the patient to obtain the device and a reduced likelihood of the patient receiving timely defibrillation treatment. The current issue is that the accessibility of AEDs varies considerably across different cities or within distinct regions of a city, which reflects spatial disparities in urban healthcare infrastructure and public health preparedness. Although there is a growing consensus on the need for more systematic and equitable AED allocation methods—such as various approaches proposed to enhance AED distribution, ranging from geographic information system (GIS)-based planning layouts (14–17) to improved site-optimization algorithms (18–20)—challenges persist. It remains unclear whether spatial deployment schemes for AEDs that solely consider distance or time factors can provide effective services for potential OHCA events. Additionally, while the objective of minimizing the distance or time required to retrieve an AED might theoretically necessitate maximizing device deployment, this approach may lead to waste in practice due to low utilization rates of the equipment.

It is common practice for decision-makers to consider the installation of AEDs in light of the relevant policy requirements (20, 21). The implementation of such installation plans is confined to specific public locations and is restricted in scope (22). It is often challenging for decision-makers to coordinate the implementation of a comprehensive installation program for a larger region. Therefore, researchers aim to provide references for decision-makers in AED planning and layout by predicting potential high-risk locations for OHCA or identifying factors influencing its occurrence. Factors such as historical OHCA case locations, population density, zones with high population mobility, and the proportion of older population have been repeatedly documented in the literature as critical determinants influencing the prediction of OHCA occurrence sites (12, 16, 17, 23). Related studies have constructed complex models based on above factors for AED siting and optimal placement. Nonetheless, existing studies on AED deployment have predominantly focused on site selection, emphasized algorithmic innovation (16, 20, 24), and overlooked the significance of identifying potential OHCA risk zones. Moreover, there is a paucity of research on constructing comprehensive predictive models for potential OHCA high-risk zones that integrate multiple factors, which could offer technical support for policymakers in large-scale AED deployment and facilitate direct replication in other cities. There is a clear need to develop a comprehensive and user-friendly technical framework for AED deployment, which can be universally applied to support policymakers throughout the entire AED spatial deployment process, from initial problem identification to subsequent research analysis and final implementation.

This study will take a variety of approaches, including understanding the current state of AED deployment and developing a spatial deployment strategy for AEDs. Specifically, this study aims to construct a multi-source data-driven priority zoning framework for AED deployment in the central urban area of Shanghai, with the sole, life-saving-oriented core objective of maximizing the coverage of potential OHCA incidence events. Our framework is designed to systematically resolve the classic trade-off between spatial coverage and incidence coverage in AED deployment, and provide scientific, replicable decision support for the optimal allocation of limited urban public health emergency resources.

## 2 Materials and methods

### 2.1 Study area and data sources

#### 2.1.1 Study area

Shanghai, China’s financial services center and a typical metropolitan area, was used as the empirical study area for the strategy proposed in this study. The area within the Shanghai Outer Ring Expressway is generally considered to be the city center of Shanghai, with a dense population and abundant public service resources. The geographic area of study area is estimated to be 664.45 km^2^, with a resident population of 11.47 million, which represents half of the total resident population (about 25 million) on one-tenth of the total land area of Shanghai.

#### 2.1.2 Data sources

In this study, the location of the AEDs was obtained by writing a Python web crawler on the WeChat app, Amerson AED Map and Jiuxin AED First Aid Map. These two maps record information on 2,265 AEDs in Shanghai, among which 963 are located in the empirical area of this study. According to the information provided by the maps, 539 devices were installed before 2021, and the remaining 424 are newly added devices by the end of 2023. The year of installation was identified by dividing the AEDs according to their code numbers.

The locations of medical institutions and various public places were obtained from the latitude and longitude by using Gaode Maps (https://ditu.amap.com/). The data with missing were screened and deleted. All latitudes and longitudes were converted to a uniform geographic coordinate system GCS _WGS_1984.

Population data were obtained from a 100 m resolution population distribution product based on the simulation of China’s seventh census (2020) (25). The percentage of population aged 60 years and above at the street level of the China’s 7th Census was used to obtain a map of the distribution of the older population in the study area at 100 m resolution.

The heights of building data were obtained from the Chinese 10 m resolution building height raster dataset (2020) produced by the GC3S team at Fudan University (26).

### 2.2 Spatial deployment strategy for AEDs

This study employs a three-step strategy to enhance the accessibility of AEDs in metropolitan areas. The approach is structured according to the “evaluate-research-apply” framework, which guides the assessment of current conditions, the exploration of potential solutions, and the implementation of effective practices. First, the spatial distribution of AEDs in the study area was clarified, and the current accessibility of AEDs was assessed. Second, a multi-factor-driven geographic information model for predicting potential OHCA risk zones was constructed, aiming to determine the priority locations for AED deployment based on risk levels. Finally, a phased AED spatial deployment plan was tailored for the study area, with the highest priority zones being addressed first.

#### 2.2.1 Step 1: Methodology for the current status evaluation

In the context of emergency medical treatment, relevant studies have indicated that the mortality rate of OHCA patients decreases significantly if they receive early defibrillation within 3–4 min (27, 28). Consequently, the “golden 4-Minute” starting from the moment an OHCA patient collapses has been established as the optimal treatment window. Integrating this with the definition of AED accessibility in this study, the service radius of AED devices can be delimited as the maximum distance that can be covered within the “golden 4-Minute.” If a patient collapses within this service range, they are considered to have relatively easy access to treatment, indicating a higher level of AED accessibility at that location.

To understand the current status of AED accessibility in a certain area, this study needs to assess the proportion of the zone falling within the service range of all AED devices, which indicates higher AED accessibility in these zones. Meanwhile, it is also necessary to calculate the number of people within the service range of all devices. This approach serves to measure the deployment efficiency of AEDs: the larger the population covered by the device service range, the more likely the current AED deployment is to cover the potential OHCA population. Regarding the maximum service radius of the devices, studies have indicated that the average speed of lay rescuers reaching a suspected OHCA location ranges between 1.8 and 2.3 m/s (29). Within the time constraint of the “golden 4 min,” the service radius of AED devices thus falls between 216 and 276 meters. For this study, a 250 m Euclidean (straight-line) distance was selected as the service radius of AEDs, rather than road network-based distance, for three key reasons aligned with the clinical reality of OHCA and our research objectives: (1) The 4-min golden treatment window creates an ultra-short, time-critical scenario where bystanders prioritize the shortest possible path over formal road networks; (2) Road network data has inherent gaps in pedestrian paths within residential complexes, commercial buildings and public places, which would create artificial accessibility blind spots in our metropolitan-scale assessment; (3) Euclidean distance provides a stable, replicable metric for cross-city application of our deployment framework, without reliance on region-specific road network data quality. The 250 m radius also includes a safety margin to account for path deviations between straight-line and actual walking distance, ensuring the 4-min treatment window is reliably met.

In this study, the “Create Buffer” tool in ArcGIS desktop software (ESRI, Redlands, CA, http://www.esri.com/), was employed to create a circular buffer centered on each device, delineating its service area. The geographic area coverage rate and population coverage rate for evaluating the current status of AED accessibility in the study area were calculated using the Areal Statistics and Spatial Join tools in ArcGIS software.

#### 2.2.2 Step 2: Methodology for identifying priority zones for AEDs deployment

By integrating data on population distribution, medical services, and urban built environment, this study constructs a geographic information model to predict risk zones for OHCA events. High-risk zones are identified as priority zones for AED deployment. This subsection provides a detailed description of the methodology employed in the selection of elements, element integration and modeling, and the hierarchical categorization of results.

##### 2.2.2.1 Selection of elements

In this study, we selected four core elements to construct the OHCA risk prediction model, with explicit definition of the correlation direction between each element and the Regional Priority Index (RPI). A higher RPI value indicates higher potential OHCA risk and higher AED deployment priority), as detailed below:

Population distribution (PD): Positive correlation with RPI. Studies have shown that the older population is a high-incidence group for OHCA events (4, 30), and their spatial distribution is significantly correlated with AED deployment (12, 16, 31). The higher the number of residents aged 60 and above in a region, the higher the potential OHCA risk and the greater the demand for AEDs. Land use type can also be used to optimize the placement of AED resources (12, 16), but it was not included in this study. Residential land use, the primary activity space for people over 60, is the core determinant of this strata’s spatial distribution, and our 100 m resolution 60 + population dataset has integrated land use type as a core covariate during its simulation (32). Adding land use type as a separate element would thus lead to severe multicollinearity with our population distribution indicator.

Medical services (MS): Negative correlation with RPI. Medical institutions are generally equipped with defibrillation devices, which can provide timely treatment for OHCA patients collapsing near them. Therefore, if a zone is rich in medical institutions, the demand for additional AED devices in that zone is correspondingly reduced. For this reason, the number of medical facilities in the zone is used as a basic element in constructing the model, with a negative correlation with AED deployment priority.

Urban built environment: Heights of Building (HB) and the number of public places (PP) were selected as components of the model, both with positive correlation with RPI.

Heights of Building (HB): Previous studies on the spatial allocation of AED resources have seldom considered vertical urban spatial factors (15, 20), or have only conducted site selection and distribution on a horizontal plane. In metropolises such as Beijing, Shanghai, Tokyo, and Hong Kong, a considerable number of high-rise residential and commercial service office buildings have been constructed, resulting in a significant concentration of population in the vertical space of these cities. Prior research has demonstrated that the number of OHCA incidents is higher in residential and office areas than in public spaces (12, 14, 33), as the general population spends the majority of their daily time in these settings. Given the continuous increase in the height of high-rise buildings, especially office and residential structures, it is reasonable to assume that these venues have a higher probability of potential OHCA occurrences, and higher demand for AED deployment. Therefore, this study incorporates the heights of buildings as a factor in the model construction.

Number of public places (PP): Public places, especially those with high population mobility and aggregation, are core priority scenarios for public-access AED deployment (12, 14, 20). OHCA events in these locations have significantly higher bystander witness rates and greater potential for early defibrillation intervention, meaning public-access AEDs can deliver the maximum life-saving benefit in these settings. The higher the number of public places in a region, the higher the potential OHCA risk and AED deployment demand. This study obtained the specific geographic locations of public places, including schools (secondary schools, universities, vocational colleges), transportation hubs (subway stations, train stations, waiting halls, large bus stations), sports and leisure venues (swimming pools, ball courts and other large sports facilities), shopping areas (supermarkets, commercial malls and other large shopping plazas), and factories. These public places were also identified by policymakers as locations with high demand for AEDs (21).

##### 2.2.2.2 Element integration and modeling

First, the hexagonal grid is used as a basic unit for element integration, and it has been extensively utilized to examine the spatial heterogeneity of urban areas (15). Under the perspective of system science, the modern city is a complex open system, and the uniform and continuous hexagonal grid can clearly express the regional heterogeneity of various factors affecting the occurrence of OHCA events (34). Therefore, the hexagon grids with higher potential OHCA risks may become candidate locations for AED deployment. To effectively implement the spatial deployment plan for AEDs, this study set the side length of the hexagon grid to 250 meters, representing the service radius of the device. Thus, the centroid of each hexagon grid can be used as a candidate location for the device.

Second, PD, MS, HB, and PP datasets were preprocessed based on hexagonal basic units. PD: the number of persons aged 60 and over in each basic unit. MS: the total number of medical institutions within each basic module. HB: the height of buildings within each basic unit. If more than half of the buildings in the basic unit are high-rise buildings, the average height of all high-rise buildings is taken; otherwise, the average height of all building heights in the basic unit is taken. PP: the total number of public places in each basic unit according to the above categorization of public places.

All data sets were sampled to the base unit and then normalized separately to eliminate the effect of magnitude differences between multi-source data. For the three positive indicators (PD, HB, PP) that are positively correlated with RPI, we adopted the standard min-max normalization method; for the negative indicator (MS) that is negatively correlated with RPI, we adopted the reverse min-max normalization method. This preprocessing step ensures all normalized indicators are positively aligned with RPI, maintaining the logical consistency of the subsequent weighted summation model: the higher the normalized value of each indicator, the greater its contribution to the final RPI, and the higher the AED deployment priority of the region. The normalization methods are as follows:

For positive indicators (PD, HB, PP)

$$X^{*}=\frac{X-X_{\min}}{X_{\max}-X_{\min}}$$ (1)

For negative indicator (MS)

$$X^{*}=\frac{X_{\max}-X}{X_{\max}-X_{\min}}$$ (2)

where $X^{*}$ is the normalized value of element, X represents the original value, $X_{\max}$ and $X_{\min}$ are the maximum and minimum.

Finally, weighted summation was used for modeling. To avoid the bias of single subjective or objective weighting methods, we adopted a widely validated combined weighting framework integrating the Analytic Hierarchy Process (AHP, subjective weighting) and the Entropy Weight Method (EWM, objective weighting) (35, 36). The complete calculation process is as follows, with full intermediate results and validation details presented in Supplementary material 1.

*Step 1*: Calculate subjective weights ( $w_{a}$ ) via AHP

AHP is a standard subjective weighting method that quantifies the relative importance of indicators based on theoretical logic and empirical evidence, following the 1–9 scale method for pairwise comparison. We first constructed a hierarchical structure with the target layer (AED deployment priority) and the criterion layer (four core indicators: PD, MS, HB, PP). Then we established a pairwise comparison judgment matrix based on the theoretical importance of each indicator for OHCA risk prediction and AED deployment demand, and conducted a strict consistency test to ensure the rationality of the judgment matrix (Consistency Ratio CR = 0.0503 < 0.1, which meets the requirement of passing the consistency test). The subjective weight $w_{a}$ of each indicator was calculated via the eigenvector method, with full details in Supplementary material 1A.

*Step 2*: Calculate objective weights ( $w_{b}$ ) via EWM

EWM is an objective weighting method that determines the weight of an indicator based on its degree of data dispersion: the greater the discrete degree of an indicator, the more information it carries, and the higher its weight. We used the normalized decision matrix (*n* = 4,240 hexagonal grid samples, m = 4 indicators) to calculate the entropy value, difference coefficient, and final objective weight $w_{b}$ of each indicator in turn, with full calculation steps in Supplementary material 1B.

*Step 3*: calculate final combination weights ( $w^{*}$ )

The combination weights were given by a correction function, calculated as in Equation 3:

$$w^{*}=\beta w_{a}+(1-\beta )w_{b}$$ (3)

where $w^{*}$ is the combination weight, $w_{a}$ is the weight calculated by AHP, and $w_{b}$ is the weight calculated by the entropy weight method. β is a constant coefficient, and in order to balance the results of the two weighting methods, *β* = 0.5. The final combination weights of the four indicators are fully presented in Supplementary material 1C.

##### 2.2.2.3 Hierarchical categorization of results

According to Equation 1–3, the model can be finally formulated as Equation 4:

$$\text{Regional priority index}\;(\mathrm{RPI})=w_{1}^{*}\mathrm{PD}+w_{2}^{*}\mathrm{MS}+w_{3}^{*}\mathrm{HB}+w_{4}^{*}\mathrm{PP}$$ (4)

where $w_{1}^{*}$ , $w_{2}^{*}$ , $w_{3}^{*}$ , and $w_{4}^{*}$ correspond to the combined weights of PD, MS, HB, PP, respectively.

The RPI was graded using the natural breaks classification (NBC) (37). The NBC is a way to find the best grouping by minimizing the within-class variance and maximizing the between-class variance. In this study, the smaller the RPI, the lower the potential OHCA risk.

#### 2.2.3 Step 3: Customized phased spatial deployment plan for AEDs

Before implementing the phased spatial deployment plan, it is necessary to validate the accuracy of the model in predicting OHCA risks. First, the NBC method is used to divide the study area into four types of zones based on the RPI, namely priority deployment zones, second-stage deployment zones, third-stage deployment zones, and non-recommended deployment zones. Next, since the base year for the model’s fundamental elements is 2020, which corresponds to the year when AED devices installed before 2021 were deployed, the locations of devices installed before 2021 can be used to validate the model’s predictive effect on potential OHCA risk zones. The higher the number of pre-2021 installed devices located in potential OHCA high-risk zones, the more reliable the model’s results can be verified.

In the four types of zones classified in this study, zones where AED devices had been installed before 2021 were excluded, and the remaining zones were those with potential OHCA risks but without AED service coverage. Therefore, a phased spatial deployment plan can be carried out according to priority. After deploying devices in each phase, the geographic coverage rate and population coverage rate of the newly added devices were evaluated respectively, and compared with the service coverage effect of the 424 devices installed in real-world scenarios after 2021.

## 3 Results

### 3.1 Spatial distribution and accessibility of AEDs

**Figure 1:**
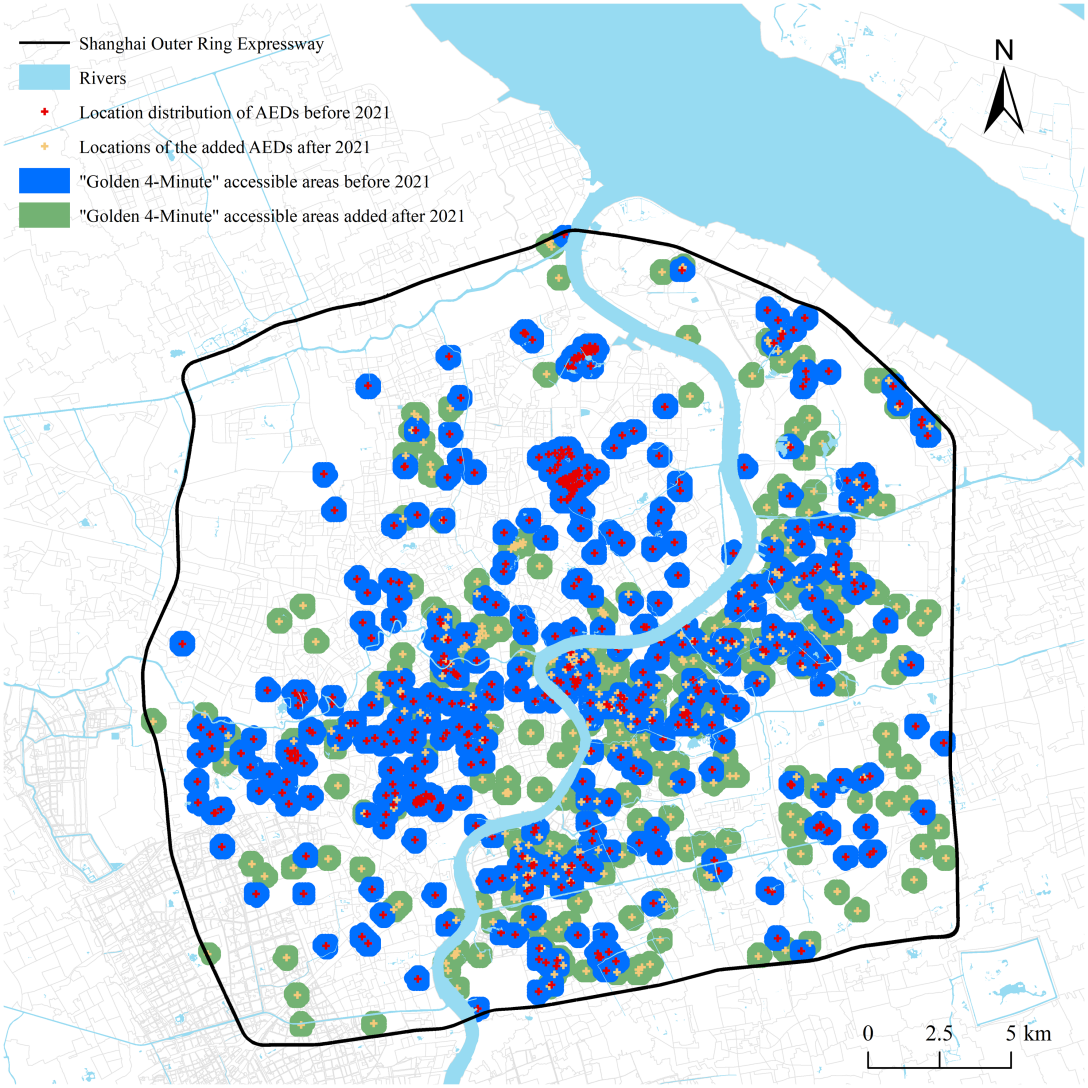
Spatial locations of AEDs installed before 2021 and the added AEDs after 2021 with their “golden 4-minute” accessible areas.

Before 2021, a total of 539 AEDs were installed in the study area. Figure 1 shows that most AED devices were installed in the center of the study area, which are the central urban areas of Shanghai with a high economic level. The blue zones in Figure 1 represent the coverage areas of AED devices within the “golden 4-Minute”, where OHCA events occur and patients can receive defibrillation treatment within 4 min. The cumulative area of the blue zones is 176.922 km^2^, accounting for 26.627% of the total area of the study area; the cumulative population in the blue zones is 4.103 million, accounting for 35.82% of the total population of the study area.

### 3.2 Identifying priority zones for AEDs placement

#### 3.2.1 Pearson correlation among the factors used to construct the model

Before conducting the correlation analysis, we first present the descriptive statistics of the four core model components (raw data before normalization, *n* = 4,240 hexagonal grid units) to clarify the data scale, distribution range and basic statistical characteristics of each indicator, as shown in Supplementary Table S2. The maximum and minimum values define the complete value range of each factor, which is the direct basis for the min-max normalization and reverse min-max normalization in our model construction.

Table 1 presented the Pearson correlation coefficients among the construction factors of the AED priority allocation model. The correlation coefficients between each pair of factors ranged from 0.1747 to 0.3071. Generally, a correlation coefficient of less than 0.3 is considered to indicate a weak correlation between two factors (15). This finding indicated that PD, MS, HB, and PP independently influenced the model results with no severe multicollinearity, and these factors are fully suitable for the construction of the model.

**Table 1:** Pearson correlation coefficients of the factors (n = 4,240).

Factors	*PD*	*MS*	*HB*	*PP*
*PD*	1			
*MS*	0.2538	1		
*HB*	0.2602	0.1747	1	
*PP*	0.2602	0.2077	0.3071	1

**Figure 2:**
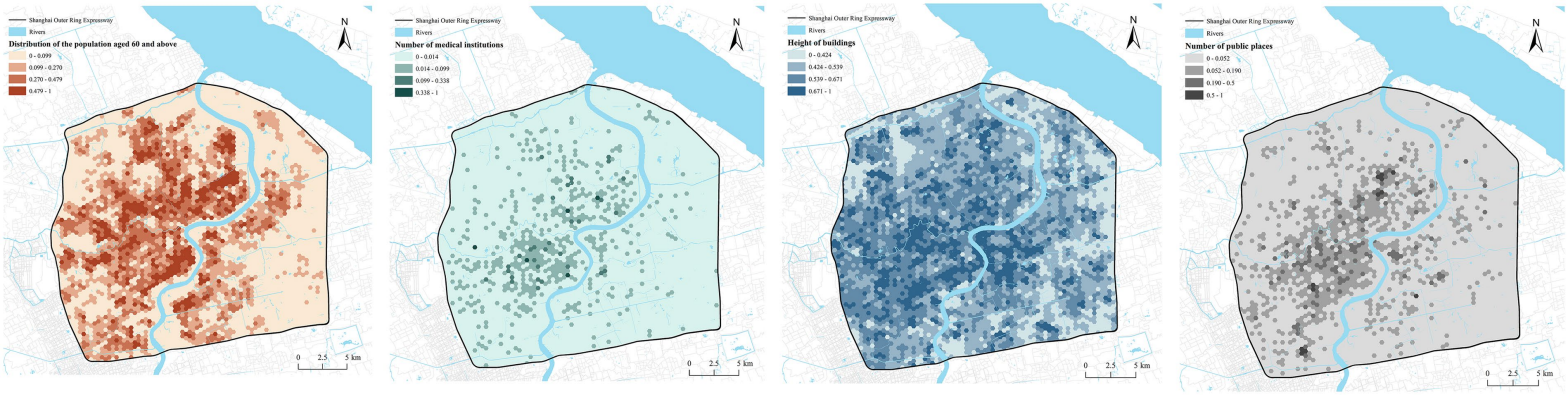
Spatial distribution of each component factor in the model. From left to right: distribution of the population aged 60 and above, number of medical institutions, height of buildings, and number of public places.

Figure 2 demonstrated the spatial distribution of the model’s component factors. As shown in Figure 1, the central urban area of Shanghai the core region of the study area not only has a higher density of AED installations but also boasts a significantly larger population of senior citizens, medical facilities, high-rise buildings, and public service amenities compared to the suburbs. Therefore, the central area of the study area is a region with high population mobility, large demand for AED devices, and higher potential OHCA risk.

#### 3.2.2 Model for identifying priority zones for AEDs deployment

Equation (4) was employed to ascertain the weights of the four normalized spatial factors (PD, MS, HB, and PP) in the model, resulting in the final formula:

RPI=PD∗0.1872+MS∗0.3748+HB∗0.2265+PP∗0.2114.

Notably, the MS indicator in this formula is the reverse-normalized value, which retains the core negative correlation between the original number of medical institutions and AED deployment priority: a higher original number of medical institutions corresponds to a lower reverse-normalized MS value, which contributes less to the final RPI and results in a lower AED deployment priority, fully consistent with the practical logic of emergency resource allocation.

**Figure 3:**
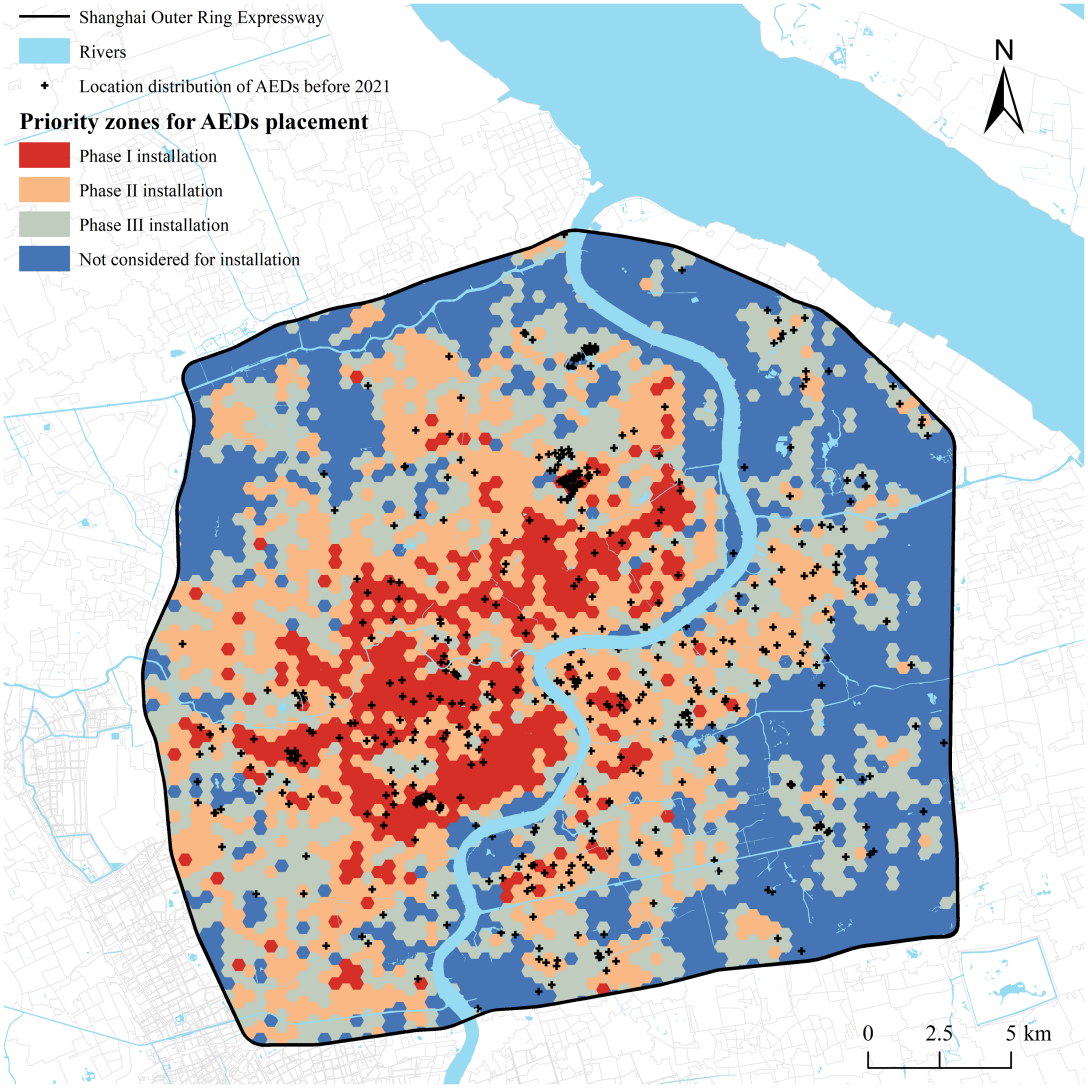
Priority zones for AEDs deployment.

The classification results of the study area obtained by the NBC method are shown in Figure 3. It can be found that the zones with the highest priority in the deployment plan are mainly distributed in the central urban areas of Shanghai. In addition, the result shows that the zones with the lowest priority are mainly distributed at the edge of the study area, that is, the suburbs. Combined with Figure 2, the number of senior citizens, high-rise buildings and public places in the suburbs is much less than that in the central urban areas. Therefore, it is suggested that decision-makers should not carry out large-scale deployment actions in the zones with the lowest priority, so as to reduce the waste of emergency medical equipment caused by too low demand.

#### 3.2.3 Model validation

There were overlaps between the spatial locations of installed AEDs by 2021 and the zones of recommended deployment calculated by the model. Table 2 showed that 92.02% of the 539 installed AEDs were located within the model-identified deployment zones for the three phases recommended for AEDs, each of which accounted for approximately 30% of the installed devices. In Figure 3, the majority of installed AEDs fell within the zones recommended by the model. These results validated the accuracy of the model’s calculations, and indicated that our multi-factor model can fully cover the reasonable dimensions of real-world experience-based AED deployment, while supplementing key factors ignored in practical decision-making.

**Table 2:** Number of existing AEDs in the zones recommended for priority installation.

Zones	*Zones of Phase I*	*Zones of Phase II*	*Zones of Phase III*	*Total*
*Number of AEDs installed*	157 (29.13%)	176 (32.65%)	163 (30.24%)	496 (92.02%)

It is necessary to clarify that pre-2021 AED deployments in Shanghai did not use any complex algorithmic model, but were based on scattered, policy-guided empirical site selection. The 92.02% matching degree is not a simple replication of existing decision-making, but a verification of the model’s reliability: our framework transforms non-replicable empirical decision-making into a standardized, universally applicable multi-factor system, which not only aligns with practical deployment logic, but also achieves significantly higher deployment efficiency than the real-world scenario. Notably, urban AED deployment decisions are fundamentally guided by the spatial distribution of OHCA incidence risk, and this real-world validation further confirms that our selected variables have strong explanatory power for identifying OHCA high-risk zones.

To verify the independent contribution of each selected variable and the robustness of our 4-factor integrated model, we conducted two sets of strict tests, based on the confirmed mutual independence of the four variables (pairwise Pearson correlation coefficients <0.3, no multicollinearity, Table 1).

First, single-variable matching tests showed that the matching degree between pre-2021 AED deployments and priority zones identified by any single variable alone (ranging from 16.33% to 64.19%) was significantly lower than the 92.02% matching degree of our integrated model. Even the two core indicators used in real-world experience-based deployments (public places and older population distribution) had a matching degree about 50 percentage points lower than our model (Supplemental materials 3A).

Second, leave-one-out sensitivity analysis further confirmed the irreplaceable incremental value of each variable: removing any single variable led to a significant drop in the model’s matching degree (from 92.02 to 49.72–61.97%), verifying that each variable provides unique information not covered by other factors (Supplemental materials 3B). The real contribution of each variable has been fully quantified via the combined AHP-entropy weight method in the model construction.

### 3.3 Phased deployment plan for AEDs

In real-world scenarios, following the year 2021, an additional 424 devices were installed in Shanghai. Figure 1 illustrated a notable expansion in the number of AEDs situated in peripheral regions distant from the city center. Compared with the service coverage of AED devices before 2021, the geographic coverage rate and population coverage rate of the newly installed 424 devices after 2021 increased by 12.61 and 12.80%, respectively, (Table 3).

**Table 3:** Compare the geographic area and population covered by AED devices in the three deployment phases with the real-world scenario.

Phase	*Phase I*	*Phase II*	*Phase III*	*Real-World scenarios*
Number of AEDs planned for installation	372	977	1,020	424
Adjusted number of AEDs planned for installation	241	433	261	-
Areas (km^2^)	257.56 (+12.14%)	426.50 (+25.43%)	555.84 (+19.47%)	260.71 (+12.61%)
Population	6,656,030 (+22.27%)	9,993,903 (+29.11%)	11,061,956 (+9.32%)	5,570,166 (+12.80%)

Areas, Population: The total geographical area or the total population that can be served by the AEDs newly installed in this phase and those already installed in the previous phase. The percentage in parentheses represents the proportion by which the geographical area and population covered by all devices increase after the installation of AEDs in this phase compared to the previous phase.

**Figure 4:**
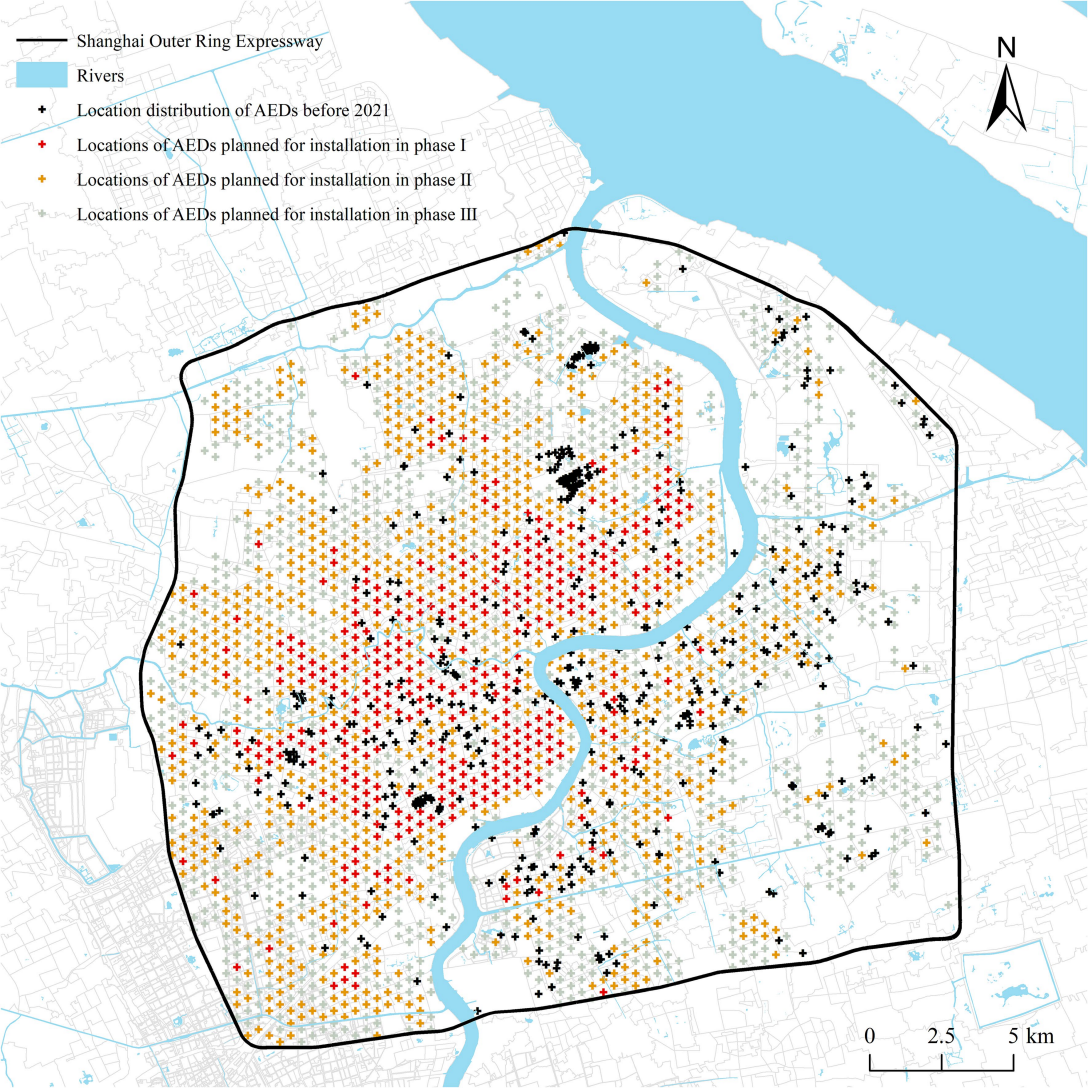
Locations of AEDs planned for installation in the three phases.

**Figure 5:**
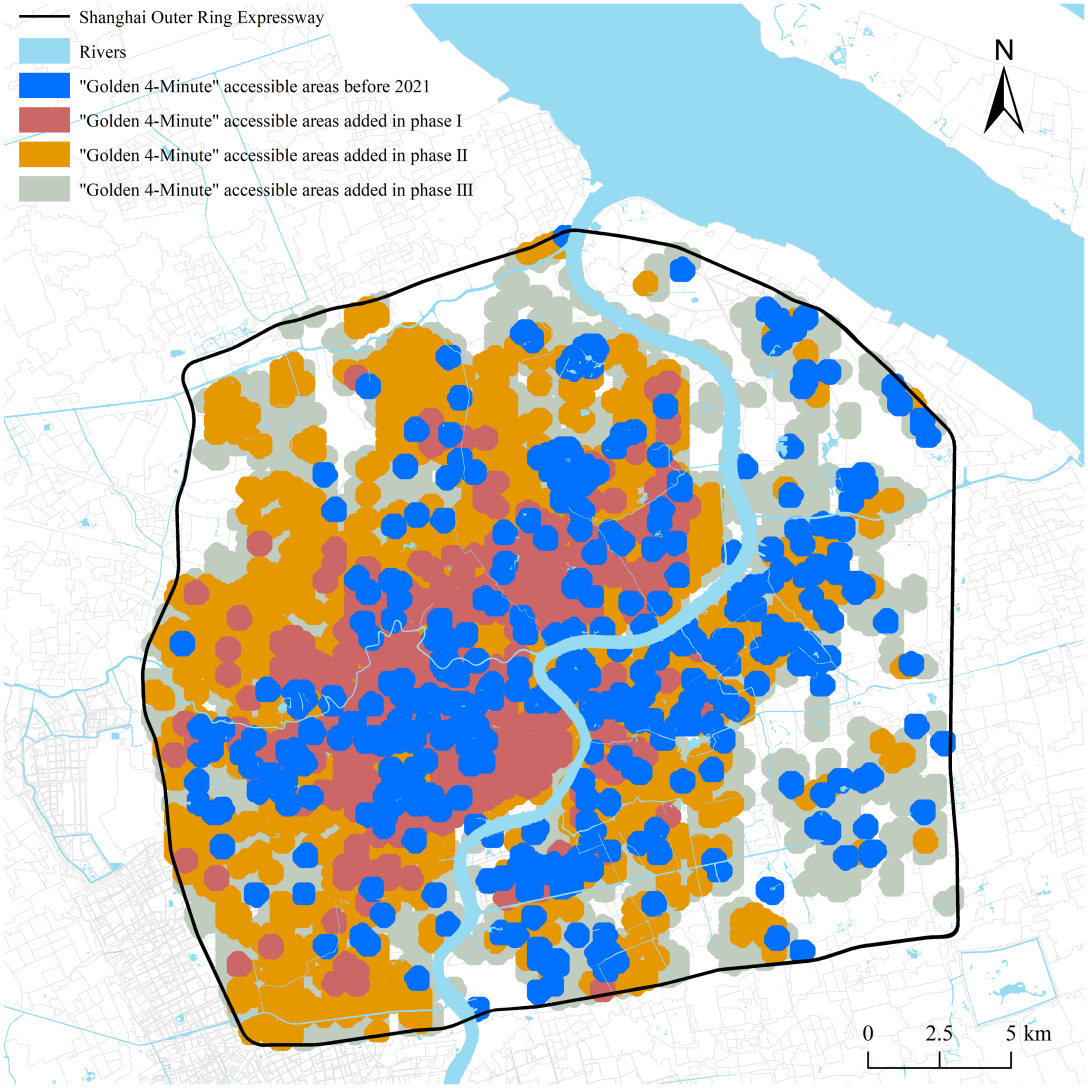
The “Golden 4-Minute” accessible areas.

A three-phase implementation of the AED deployment program was recommended based on the model results (Figure 4). Table 3 showed the minimum number of devices required to be installed in each phase and the coverage effect of emergency services provided after installation (Figure 5). After implementing the first phase of the installation plan, these devices could increase the geographic coverage area by 12.14%, which was comparable to the results after installing 424 devices in real-world scenarios. It is of greater significance that the Phase I deployment program resulted in a 22.27% increase in population coverage, which is approximately 10% higher than that of a device deployed in a real scenario.

Since the new accessible area of the devices formed after the completion of the deployment plan of the previous phase may cover the location of some of the devices to be deployed in the later phase, so we excluded the devices that were planned to be deployed in the later phase and were located within the accessible area of the previous phase, and thus got the Adjusted number of AEDs planned for installation (Table 3).

The first phase of the deployment plan achieved almost the same new coverage area as the real-world scenario (12.14% vs. 12.61%), but the number of recommended installations in the first phase was smaller than that in the real-world scenario (241 vs. 424), and it also covered more people with potential needs. This result indicated that the model-driven AED installation strategy was more efficient. The second phase of the installation plan increased the coverage area and population more than the first phase, and after the plan was implemented, 87.17% of the population was covered within the service area of AEDs, a figure that reached 96.49% after the completion of the third phase plan.

## 4 Discussion

Prior to proposing a phased deployment plan, it is essential to ascertain the service radius of the AEDs, as this information is instrumental in determining the requisite scale of the equipment deployment. In this study, a linear-based model (38) was employed to illustrate the service area provided by the devices with a radius of 250 meters. This approach strikes a balance between the simplicity of the model construction and the precision of the evaluation of device accessibility. On the basis of the above work, through evaluating the current situation, constructing a multi-factor comprehensive model, and then formulating a phased deployment plan, this study proposes a full-cycle spatial deployment strategy for AED devices. For decision-makers, these strategies are more beneficial than attempting to determine the exact installation locations of devices, as site selection studies (20, 24) may overlook the real-world constraints of device installation. For example, in indoor spaces such as subway stations, it is difficult to determine the exact location of AEDs.

When historical information on OHCA events is unavailable, the use of geographic information technology to construct a risk prediction model guided by a comprehensive index can effectively identify zones at high risk for potential OHCA incidents and guide the optimization of AED spatial layout and implementation of installation plans (15). In contrast with the extant practice of AED spatial allocation (15, 17, 20), which employed factors such as mobile population, traffic route density, and land use to construct the model, this study considered the following aspects in the model construction process: (1) The service radius of AED resources is limited, and there are rigorous requirements regarding the time required to obtain the device. Consequently, individuals may not necessarily traverse the typical route of travel to obtain AED resources (38). The extent to which high-grade roads, such as expressways, enhance accessibility to AED resources within the narrow window of opportunity (approximately 4 minutes) during which a cardiac arrest patient may benefit from such resources remains to be demonstrated. Consequently, to enhance the real-world accessibility of AEDs in the 4-min emergency window, this study did not incorporate the road network as a factor in constructing the model. The Euclidean distance-based circular buffer avoids overestimating AED coverage from optimal road network paths, eliminates accessibility blind spots from incomplete pedestrian network data, and ensures our deployment strategy prioritizes the actual availability of AEDs during OHCA events, rather than map-based theoretical reachability. (2) The construction of the model in this study took into account the impact of building elevation. However, the model’s results did not provide a plan for the vertical installation of equipment in the final three-phase device installation plan, which represents a limitation of this study. In addition to considering the service effect of AED resources in the horizontal direction, it is also important to maximize the coverage of AEDs in the vertical direction in the future, especially in densely populated areas with high-rise buildings in metropolises.

It is necessary to clarify the core differences and applicable boundaries between our proposed multi-source data-driven deployment framework and classic point-level location optimization models (e.g., p-median) that are widely used in facility siting research. Generic optimization models represented by p-median are designed to solve a well-defined fixed-point siting problem: given a pre-determined number of facilities and static demand points (typically geocoded historical OHCA incident locations), they output the optimal set of specific locations to minimize the overall access cost for end users (19). In contrast, our framework is developed to address the unmet practical needs of metropolitan-scale, phased AED deployment, with three core distinctions: (1) our core output is regional priority zoning and a phased implementation roadmap, rather than a fixed set of specific siting points; (2) our framework does not rely on historical OHCA data, making it replicable and scalable in regions where such high-quality data are unavailable; (3) the deployment scale for each phase in our plan is dynamically calculated based on priority grading and existing coverage overlap elimination, rather than relying on a pre-fixed total number of devices.

These two methodological systems have complementary rather than competitive value in full-cycle AED deployment practice. Our priority zoning framework provides decision-makers with a macro-level, phased planning strategy to optimize resource allocation across large urban areas and avoid wasteful investment in low-risk zones, while classic location optimization models can be further applied to conduct fine-grained point-level siting within the high-priority zones identified by our framework. For the core research objective of this study, to construct a full-cycle, universally applicable deployment strategy for metropolitan AED rollout, the effectiveness of our framework is sufficiently validated by real-world deployment data, rather than cross-model comparison with point-level optimization tools that address a distinct scientific question.

A core design of our framework is to resolve the inherent conflict between maximum spatial coverage and maximum OHCA incidence coverage in AED deployment. For high-risk, high-density core urban areas, we prioritize incidence coverage maximization, allowing reasonable overlap of AED service scopes to meet the high-density emergency demand, cope with simultaneous OHCA events, and reduce vertical access time in high-rise buildings. For low-risk, low-density urban fringe areas, we prioritize the cost efficiency of resource investment, using the minimum number of devices to achieve basic spatial coverage, to avoid the resource waste caused by blind pursuit of incidence coverage in areas with extremely low OHCA risk. This hierarchical design ensures that limited AED resources are allocated to the areas with the highest life-saving value first, maximizing the overall emergency benefit of the deployment plan. Our model allows reasonable overlap of AED service scopes in high-risk, high-density areas to maximize incidence coverage, but does not conduct fine-grained optimization of the specific number of devices in a single unit to avoid excessive overlap. This fine-grained single-point optimization can be carried out on the basis of our priority zoning framework in future practical deployment.

The incidence of OHCA events may exhibit seasonal variations (39), and the dynamics of population distribution (15) could also influence the deployment of AEDs. However, this study does not account for the spatiotemporal heterogeneity of OHCA events across different seasons and physical spaces, which also represents a limitation. Another limitation is that our Euclidean distance-based accessibility assessment may overestimate reachability in geographically isolated areas (e.g., river-separated zones, enclosed industrial parks) and indoor vertical spaces. While our 250 m service radius includes a safety margin to mitigate this bias, future fine-grained site-specific optimization should integrate high-precision pedestrian network and indoor path data to refine deployment plans for these special scenarios. In addition, we did not include fine-grained socioeconomic indicators (e.g., income level) in our model to ensure the universal replicability of our framework, as such high-resolution spatial data are not widely available in most global cities; these indicators can be incorporated for localized optimization in future region-specific studies. Due to the unavailability of fine-grained, geocoded OHCA incidence data in our study area, we did not conduct regression analysis to verify the independent explanatory power of each variable against actual OHCA incidences. For future localized optimization of the framework in cities with available OHCA incidence data, regression-based variable validation and weight calibration can be further implemented to improve the model’s localized performance.

## 5 Conclusions and recommendations

### 5.1 Conclusion

This study proposes a comprehensive spatial deployment strategy for AEDs in metropolitan areas, which consists of three steps: current status assessment, multi-factor modeling, and phased planning. The research framework, tested in Shanghai’s central urban area, yields three key findings:

First, the multi-factor risk prediction model, incorporating older population distribution, medical facilities, building heights, and public places, effectively identifies high-risk zones for OHCA. The NBC method classifies the study area into four tiers for sequential deployment planning, confirming that central Shanghai—characterized by dense older populations, high-rise buildings, and public facilities—exhibits higher OHCA risks than the suburbs. Model validation shows that 92.02% of AED installations prior to 2021 align with the zones recommended by the model, highlighting its reliability.

Second, the phased deployment plan demonstrates superior efficiency compared to real-world scenarios. The first phase, recommending 241 AED installations, achieved a 12.14% increase in geographic coverage and a 22.27% increase in population coverage—comparable to the real-world 424-device deployment but with 43% fewer devices. By the third phase, the plan could cover 96.49% of the population, far exceeding the pre-2021 baseline of 35.82%. This highlights the model’s potential to optimize resource allocation and reduce equipment waste in low-risk suburbs.

Third, the study reveals spatial disparities in AED accessibility. The “golden 4-Minute” coverage area before 2021 encompassed only 26.63% of the study area, emphasizing the urgent need for strategic expansion. The model-driven approach prioritizes zones where AEDs can be accessed within 250 meters, aligning with the critical window for effective defibrillation.

### 5.2 Policy implications and recommendations

#### 5.2.1 Targeted prioritization in high-risk zones

Decision-makers should prioritize AED deployment in central urban areas identified by the RPI model, where older populations, high-rise buildings, and public places converge. Suburbs with low RPI scores require minimal investment to avoid resource waste, as validated by their lower OHCA risk and existing coverage gaps.

#### 5.2.2 Phased implementation for efficiency

Adopt the three-phase deployment strategy to incrementally expand coverage. Phase I should focus on densely populated hubs, followed by systematic expansion to secondary zones. This approach ensures each installation maximizes geographic and population coverage, as demonstrated by the 22.27% population coverage gain in Phase I.

#### 5.2.3 Incorporate vertical urban dynamics

Future models should account for vertical population density in high-rise buildings, as OHCA risks may vary across floors. Collaborate with building managers to deploy AEDs in strategic vertical locations (e.g., lobby areas, elevators) to enhance accessibility in vertical spaces.

#### 5.2.4 Data-driven adaptation

Regularly update the model with real-time data on population distribution, building developments, and OHCA incidents to refine risk predictions. This flexibility is crucial for adapting to urban growth, such as new commercial districts or transit hubs.

#### 5.2.5 Integrate indoor space planning

While the study excluded road networks to prioritize short-distance accessibility, future research should collaborate with urban planners to map AED locations in indoor spaces (e.g., subway stations, malls), addressing real-world installation constraints.

## Data Availability

The raw data supporting the conclusions of this article will be made available by the authors, without undue reservation.
