# Supplementary material for: Towards efficient AED deployment: a multi-source data-driven geographic information approach
Source: Front Public Health. 2026 May 29;14:1836037. doi: 10.3389/fpubh.2026.1836037 (PMC13260465; doi:10.3389/fpubh.2026.1836037)
Supplement: Supplementary file 1 [file Supplementary_file_1.docx]

Supplementary Material

# Complete Calculation Process of Model Indicator Weights

This section presents the full, step-by-step calculation process of the subjective weights (AHP), objective weights (Entropy Weight Method), and final combination weights of the four model indicators.

**A: Calculation of AHP Subjective Weights**

**A.1 Hierarchical Structure and Pairwise Comparison Scale**

Target Layer: AED deployment priority (Regional Priority Index, RPI)

Criterion Layer: 4 core indicators: Population Distribution (PD), Medical Services (MS), Building Height (HB), Number of Public Places (PP)

Pairwise Comparison Scale: Standard 1-9 scale method (1 = equal importance, 9 = extreme importance of one indicator over another)

**A.2 Pairwise Comparison Judgment Matrix**

Based on the theoretical importance of each indicator for OHCA risk prediction and AED deployment demand, we constructed the following judgment matrix (4×4, corresponding to 4 indicators in the order of PD, MS, HB, PP):

| **Indicator** | **PD** | **MS** | **HB** | **PP** |
| --- | --- | --- | --- | --- |
| PD | 1 | 1/3 | 8 | 1/2 |
| MS | 3 | 1 | 1/8 | 1 |
| HB | 1/8 | 8 | 1 | 1/7 |
| PP | 2 | 1 | 7 | 1 |

**A.3 Consistency Test**

To ensure the rationality of the judgment matrix, we conducted a strict consistency test following standard AHP procedures:

Calculate the maximum eigenvalue of the judgment matrix: $\lambda_{max}$=4.1344

Calculate the Consistency Index (CI): $CI=\frac{\lambda_{max}-m}{m-1}$=0.0448 (where m=4, the number of indicators)

Look up the Random Consistency Index (RI) for m=4: RI=0.89 (standard value for AHP)

Calculate the Consistency Ratio (CR): $CR=\frac{CI}{CR}$=0.05033708

The consistency test result CR=0.05033708<0.1, which fully meets the academic standard for passing the AHP consistency test, confirming the rationality of the judgment matrix.

**A.4 Final AHP Subjective Weights**

We calculated the subjective weights via the normalized eigenvector method, with the final results:

| **Indicator** | **PD** | **MS** | **HB** | **PP** |
| --- | --- | --- | --- | --- |
| AHP Subjective Weight ($\boldsymbol{w}_{\boldsymbol{a}}$) | 0.2025 | 0.4021 | 0.0417 | 0.3508 |
| Sum of Weights | 1.0000 |  |  |  |

**B: Calculation of Entropy Weight Method (EWM) Objective Weights**

**B.1 Basic Parameters**

Number of samples (hexagonal grid units): n=4240

Number of indicators: m=4 (PD, MS, HB, PP)

Input matrix: Normalized decision matrix $X={(x_{ij})}_{n*m}$ (from the min-max/reverse min-max normalization in the main manuscript,$x_{ij}\epsilon[0, 1]$

**B.2 Step-by-Step Calculation**

1.Calculate the proportion of the *i*-th sample under the *j*-th indicator:

$$p_{ij}=\frac{x_{ij}}{\sum_{i=1}^{n} x_{ij}},where i=1,2,...,n;j=1,2,...,m$$

Note: For $x_{ij}$=0, we set $p_{ij}$=0 to avoid invalid calculation of the logarithm.

2.Calculate the entropy value of the *j*-th indicator:

$$e_{j}=-k\sum_{i=1}^{n} p_{ij}\ln(p_{ij}), where k=\frac{1}{\ln(n)}=\frac{1}{\ln(4240)}\approx0.1178, to ensure e_{j}\epsilon[0, 1]$$

3.Calculate the difference coefficient of the *j*-th indicator:

$$g_{j}=1-e_{j}$$

The larger the $g_{j}$, the greater the discrete degree of the *j*-th indicator, the more information it carries, and the higher its weight.

4.Calculate the final EWM objective weight:

$$w_{bj}=\frac{g_{j}}{\sum_{j=1}^{m} g_{j}}$$

**B.3 Final EWM Objective Weights**

| **Indicator** | **PD** | **MS** | **HB** | **PP** |
| --- | --- | --- | --- | --- |
| Entropy Value ($e_{j}$, raw value) | 75.2153 | 153.4959 | 181.5161 | 32.5868 |
| Difference Coefficient ($g_{j}$, raw value) | -74.2153 | -152.4959 | -180.5161 | -31.5868 |
| EWM Objective Weight ($w_{bj}$) | 0.16912 | 0.3475 | 0.4114 | 0.0720 |
| Sum of Weights | 1.0000 |  |  |  |

**step C: Calculation of Final Combination Weights**

We used the balance coefficient β=0.5 to combine the AHP subjective weights and EWM objective weights, with the formula:

$\boldsymbol{w}^{\boldsymbol{*}}\boldsymbol{=}\boldsymbol{\beta}\boldsymbol{w}_{\boldsymbol{a}}\boldsymbol{+(1-}\boldsymbol{\beta}\boldsymbol{)}\boldsymbol{w}_{\boldsymbol{b}}$

The final combination weights of the four indicators are as follows, which are applied to the RPI model in the main manuscript:

| **Indicator** | **AHP Subjective Weight (**$\text{w}_{\text{a}}$**)** | **EWM Objective Weight (**$\text{w}_{\text{b}}$**)** | **Final Combination Weight (**$\text{w}^{\text{∗}}$**)** |
| --- | --- | --- | --- |
| PD | 0.2053 | 0.169126907 | 0.187213454 |
| MS | 0.4021 | 0.347518197 | 0.374809098 |
| HB | 0.0417 | 0.411372666 | 0.226536333 |
| PP | 0.3508 | 0.07198223 | 0.211391115 |
| Sum | 1.0000 | 1.0000 | 1.0000 |

The final RPI model formula is:

RPI = PD*0.187213454 + MS*0.374809098 + HB*0.226536333 + PP*0.211391115

# Descriptive statistics of core model components (raw data before normalization)

| **Model Component** | **Measurement Unit** | **Minimum Value** | **Maximum Value** | **Mean** | **Standard Deviation** |
| --- | --- | --- | --- | --- | --- |
| Population Distribution (PD) | Number of residents aged 60+ per hexagonal unit | 0 | 31377 | 4848.80 | 5896.45 |
| Medical Services (MS) | Number of medical institutions per hexagonal unit | 0 | 71 | 0.61 | 2.04 |
| Building Height (HB) | Average building height per hexagonal unit (m) | 0 | 38.62 | 20.60 | 4.90 |
| Number of Public Places (PP) | Number of public places per hexagonal unit | 0 | 58 | 2.37 | 4.14 |

# Verification of variable real contribution

**3A. Single-variable matching tests**

We conducted a strict single-variable matching test, using the identical natural breaks classification (NBC) method and validation standard as our full model, to calculate the matching degree between pre-2021 AED deployments and priority zones identified by each single variable alone. The results are as follows:

| **Single Variable** | **Matching Degree with Pre-2021 AED Deployments** |
| --- | --- |
| Number of Public Places (PP, core indicator used in real-world deployment) | 193/539=35.807% |
| Population Distribution (PD, secondary indicator used in real-world deployment) | 233/ 539=43.228% |
| Building Height (HB) | 346/539=64.193% |
| Medical Services (MS) | 88/539=16.327% |
| Our 4-factor integrated model | 92.02% |
| These results directly confirm that no single variable can achieve a better matching degree than our multi-factor model. Even the two core single indicators used by real-world decision-makers have a matching degree more than 13 percentage points lower than our integrated model. This fully demonstrates that the high performance of our model comes from the complementary integration of all four mutually independent variables, rather than simply replicating the single-variable decision logic of real-world deployments. | |

**3B. Leave-one-out sensitivity analysis**

The results show that removing any single variable leads to a significant drop in the model’s matching degree:

1.Removing the MS variable: matching degree drops from 92.02% to 60.853% (328/539)

2.Removing the HB variable (vertical spatial factor ignored in most existing studies and real-world deployments): matching degree drops to 49.722% (268/539)

3.Removing the PD variable: matching degree drops to 61.967% (334/539)

4.Removing the PP variable: matching degree drops to 59.926% (323/539)

This analysis fully verifies that each of the four independent variables provides unique incremental information that cannot be covered by other factors, and their real contribution has been fully quantified via the combined AHP-entropy weight method in our model construction.
